# The Impact of Different DNA Extraction Kits and Laboratories upon the Assessment of Human Gut Microbiota Composition by 16S rRNA Gene Sequencing

**DOI:** 10.1371/journal.pone.0088982

**Published:** 2014-02-24

**Authors:** Nicholas A. Kennedy, Alan W. Walker, Susan H. Berry, Sylvia H. Duncan, Freda M. Farquarson, Petra Louis, John M. Thomson, Other members not named within the manuscript author list (alphabetical by surname):, T Ahmad, CA Anderson, JC Barrett, H Drummond, C Edwards, A Hart, C Hawkey, P Henderson, M Khan, CA Lamb, JC Lee, JC Mansfield, CG Mathew, C Mowat, WG Newman, NJ Prescott, A Simmons, P Simpson, K Taylor, K Taylor, DC Wilson, Jack Satsangi, Harry J. Flint, Julian Parkhill, Charlie W. Lees, Georgina L. Hold

**Affiliations:** Peninsula College of Medicine and Dentistry University of Exeter, EX1 2LU, UK; The Wellcome Trust Sanger Institute, Wellcome Trust Genome Campus, Hinxton, Cambridge, CB10 1SA, UK; The Wellcome Trust Sanger Institute, Wellcome Trust Genome Campus, Hinxton, Cambridge, CB10 1SA, UK; Gastrointestinal Unit, Centre for Genomic and Experimental Medicine, Institute of Genetics and Molecular Medicine, University of Edinburgh, Edinburgh EH4 2XU, UK; Department of Gastroenterology, Torbay Hospital, Torbay TQ2 7AA, UK; Inflammatory Bowel Disease Unit, St Mark's Hospital, Watford Road, Harrow, Middlesex HA1 3UJ, UK; Nottingham Digestive Diseases Centre, Queen's Medical Centre, Nottingham NG7 1AW, UK; Royal Hospital for Sick Children, Paediatric Gastroenterology and Nutrition, Glasgow G3 8SJ, UK; Department of Medical Genetics, Manchester Academic Health Science Centre (MAHSC), University of Manchester, Manchester M13 0JH, UK; Department of Gastroenterology & Hepatology, University of Newcastle upon Tyne, Royal Victoria Infirmary, Newcastle upon Tyne NE1 4LP, UK; Inflammatory Bowel Disease Research Group, Addenbrooke's Hospital, University of Cambridge, Cambridge CB2 0QQ, UK; Department of Gastroenterology & Hepatology, University of Newcastle upon Tyne, Royal Victoria Infirmary, Newcastle upon Tyne NE1 4LP, UK; Department of Medical and Molecular Genetics, King's College London School of Medicine, 8th Floor Guy's Tower, Guy's Hospital, London, SE1 9RT, UK; Department of General Internal Medicine, Ninewells Hospital and Medical School, Ninewells Avenue, Dundee DD1 9SY, UK; Department of Medical Genetics, Manchester Academic Health Science Centre (MAHSC), University of Manchester, Manchester M13 0JH, UK; Department of Medical and Molecular Genetics, King's College London School of Medicine, 8th Floor Guy's Tower, Guy's Hospital, London, SE1 9RT, UK; Translational Gastroenterology Unit, Experimental Medicine Division, John Radcliffe Hospital, Headington, Oxford OX3 9DU, UK; Translational Gastroenterology Unit, Experimental Medicine Division, John Radcliffe Hospital, Headington, Oxford OX3 9DU, UK; Translational Gastroenterology Unit, Experimental Medicine Division, John Radcliffe Hospital, Headington, Oxford OX3 9DU, UK; Department of Gastroenterology, Guy's & St Thomas' NHS Foundation Trust, St Thomas' Hospital, London SE1 7EH, UK; Child Life and Health, University of Edinburgh, Edinburgh EH9 1UW, UK; 1 Gastrointestinal Unit, Centre for Genomic and Experimental Medicine, University of Edinburgh, Western General Hospital, Edinburgh, United Kingdom; 2 Pathogen Genomics Group, Wellcome Trust Sanger Institute, Wellcome Trust Genome Campus, Hinxton, Cambridgeshire, United Kingdom; 3 Gastrointestinal Research Group, Division of Applied Medicine, Aberdeen University, Aberdeen, United Kingdom; 4 Rowett Institute of Nutrition and Health, Aberdeen University, Aberdeen, United Kingdom; 5 Department of Digestive Disorders, Aberdeen Royal Infirmary, Foresterhill, Aberdeen, United Kingdom; Instutite of Agrochemistry and Food Technology, Spain

## Abstract

**Introduction:**

Determining bacterial community structure in fecal samples through DNA sequencing is an important facet of intestinal health research. The impact of different commercially available DNA extraction kits upon bacterial community structures has received relatively little attention. The aim of this study was to analyze bacterial communities in volunteer and inflammatory bowel disease (IBD) patient fecal samples extracted using widely used DNA extraction kits in established gastrointestinal research laboratories.

**Methods:**

Fecal samples from two healthy volunteers (H3 and H4) and two relapsing IBD patients (I1 and I2) were investigated. DNA extraction was undertaken using MoBio Powersoil and MP Biomedicals FastDNA SPIN Kit for Soil DNA extraction kits. PCR amplification for pyrosequencing of bacterial 16S rRNA genes was performed in both laboratories on all samples. Hierarchical clustering of sequencing data was done using the Yue and Clayton similarity coefficient.

**Results:**

DNA extracted using the FastDNA kit and the MoBio kit gave median DNA concentrations of 475 (interquartile range 228-561) and 22 (IQR 9-36) ng/µL respectively (p<0.0001). Hierarchical clustering of sequence data by Yue and Clayton coefficient revealed four clusters. Samples from individuals H3 and I2 clustered by patient; however, samples from patient I1 extracted with the MoBio kit clustered with samples from patient H4 rather than the other I1 samples. Linear modelling on relative abundance of common bacterial families revealed significant differences between kits; samples extracted with MoBio Powersoil showed significantly increased *Bacteroidaceae*, *Ruminococcaceae* and *Porphyromonadaceae*, and lower *Enterobacteriaceae, Lachnospiraceae*, *Clostridiaceae*, and *Erysipelotrichaceae* (p<0.05).

**Conclusion:**

This study demonstrates significant differences in DNA yield and bacterial DNA composition when comparing DNA extracted from the same fecal sample with different extraction kits. This highlights the importance of ensuring that samples in a study are prepared with the same method, and the need for caution when cross-comparing studies that use different methods.

## Introduction

The last decade has seen a marked rise in interest in the bacterial communities that coexist within humans, facilitated by the availability of modern molecular techniques. The Human Microbiome Project[1] and MetaHIT[2] have made considerable progress in furthering our understanding of microbial diversity and community structure in different body areas of healthy individuals. The gastrointestinal tract is the most heavily colonized organ in the body, with 70% of bacteria found in humans residing in the colon[3]–[5]. Differences in the diversity and community structure of the gut microbiota have been associated with diseases of the gastrointestinal tract such as inflammatory bowel disease (IBD)[6], [7] and irritable bowel syndrome[8], as well as metabolic disorders like type 2 diabetes mellitus and obesity[9].

Determining the bacterial community structure in fecal samples through amplification and sequence analysis of extracted DNA has revolutionized gastrointestinal microbiology research over recent years. These culture-independent techniques for assessing diversity have largely replaced traditional culture based approaches as they are considered to be less biased in terms of defining true diversity and considerably less labor-intensive[10], [11]. Due to the recent rapid increase in DNA-based phylogenetics of bacterial communities many different DNA extraction procedures are used, each with its own potential biases. All methods rely on chemical or mechanical disruption, lysis using detergents, or a combination of these approaches.

Previous studies have evaluated differences between DNA extraction methods from fecal samples, exploring detection with conventional PCR[12], [13], quantitative PCR[14], [15], bands on denaturing gradient gel electrophoresis (DGGE)[15]–[20] and phylogenetic microarray[21]. Significant differences in relative abundance have been demonstrated when DNA was extracted using different methods from mock communities of bacteria and assessed by 16S rRNA sequencing[22], [23]. Wu *et al.* described the effect of different fecal extraction methods on 16S rRNA pyrosequencing, comparing QIAamp DNA Stool Minikit, MoBio PowerSoil DNA Isolation Kit and Stratec PSP Spin Stool DNA Kit[24].

The aim of this study was to analyze bacterial communities in healthy volunteer and IBD patient fecal samples extracted using the MoBio and FastDNA DNA extraction kits in two established gastrointestinal research laboratories. The MoBio Power Soil DNA extraction kit and the MP Biomedicals Fast DNA Spin Kit for Soil DNA extraction kit are two commonly used extraction procedures for fecal microbial diversity studies[25]–[27]. Both methods use a combination of mechanical disruption and chemical lysis.

## Methods

### Fecal sample collection and initial processing

**Figure 1:**
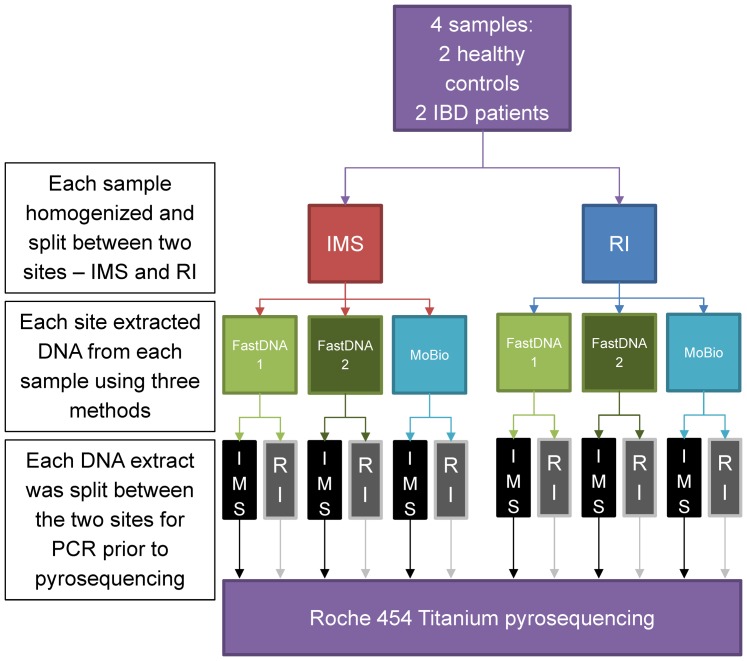
Study protocol. IMS: Institute of Medical Sciences, University of Aberdeen, Foresterhill, Aberdeen; RI: Rowett Institute of Nutrition and Health, University of Aberdeen, Bucksburn, Aberdeen.

Fecal samples were taken from two patients with IBD (I1 and I2) and from two healthy controls (H3 and H4) using the Fisher Fecal Commode Collection Kit. Fecal samples were kept at 4°C and processed within 4 hours of collection. This short period of storage is not expected to influence molecular estimation of microbial community composition[25]. Each sample was thoroughly mixed and several aliquots of 500 mg were dispensed. Aliquots were distributed between two established microbial research laboratories (Institute of Medical Sciences (IMS) and The Rowett Institute of Nutrition and Health (RINH), both Aberdeen University) and then subject to further processing as detailed in Figure 1 and described below.

### Ethics Statement

Ethical approval was granted by North of Scotland Research Ethics Service (03/0137 and 12/NS/0061) on behalf of all participating centers and written informed consent was obtained from all subjects.

### MoBio PowerSoil DNA extraction procedure

One 500 mg fecal aliquot was used for MoBio PowerSoil DNA isolation kit extraction. 5 ml of MoBio lysis buffer was immediately added to the fresh fecal sample, which was then vortex mixed for 30–40 seconds. Fecal suspensions were then centrifuged (1,500 g×5 minutes) and 1 ml of the supernatant placed into the MoBio Garnet bead tubes containing 750 ul of MoBio buffer. These tubes were then heated at 65°C for 10 minutes, then at 95°C for 10 minutes. Samples were then stored at −80°C prior to processing in both laboratories following the manufacturer's instructions. DNA was eluted in 100 µL MoBio elution buffer.

### FastDNA SPIN Kit for Soil procedure

For each fecal sample 2×500 mg aliquots were placed in FastDNA SPIN Kit lysing matrix E tubes and 978 µl of sodium phosphate buffer and 122 µl MT buffer were added to each tube and vortex mixed. One aliquot was then stored at −80°C and was defined as FastDNA method 1. The second aliquot was subjected to additional processing by heating at 65°C for 10 minutes, then at 95°C for 10 minutes followed by storage at −80°C. This was defined as FastDNA method 2. Both aliquots were then processed following manufacturer's (Qbiogene, MP Biomedicals, Illkirch, France) instructions. DNA was eluted in 100 µL FastDNA elution buffer.

### PCR amplification

Fecal DNA was quantified by Nanodrop mass spectrophotometry before dilution to 25 ng/µl. Initial PCR amplification was undertaken at each laboratory with Invitrogen AccuPrime Taq DNA Polymerase High Fidelity utilising a per-reaction mix of 2 µl of DNA template, 2 µl of Buffer II, 0.2 µl (2 µM) Fusion Primer A, 0.2 µl (2 µM) Fusion Primer B, 0.08 µl (1 U) Accuprime Taq and 15.52 µl sterile, deionized water to a final volume of 20 µl. Quadruplicate PCR reactions were set up per DNA sample. The 16S rRNA gene primers, spanning the V3-5 region of the 16S rRNA gene, were configured as follows: 338F, 5′-*CCTATCCCCTGTGTGCCTTGGCAGTCTCAG*ACTCCTACGGGAGGCAGCAG-3′, where the bases in italics are 454 Lib-L kit adaptor “B”, and 926R, 5′- CCATCTCATCCCTGCGTGTCTCCGACTCAG-Marker-CCGTCAATTCMTTTRAGT-3′, where the underlined bases are 454 adaptor “A” and the marker sequence was a unique 12-mer string of error-correcting Golay barcode bases for each sample[28]. No barcode was added to the forward primer. Hence the PCR products were flanked by a 40 bp fusion primer/multiplex identifier sequence at the reverse end and a 30 bp fusion primer at the forward end. PCR cycling conditions were as follows: 2 minutes at 94°C; 20 cycles of 30 seconds at 94°C, 30 seconds at 53°C, 120 seconds at 68°C. Following confirmation of adequate and appropriately sized product, the quadruplicate reactions were pooled and ethanol precipitated prior to purification as per the recommended AMPure purification method for 454 sequencing. The PCR products were then sequenced with the Roche 454 Titanium sequencing platform using the Lib-L kit (Wellcome Trust Sanger Institute, Cambridgeshire, UK). The sequence data are available from the European Nucleotide Archive under Study Accession Numbers ERP004371 and ERP004372, and Sample Accession Numbers ERS373486 and ERS373498. The relevant barcode information for each of the samples is shown in Table S1.

### Quantitative PCR

Quantitative real-time PCR was performed as described previously[29]. Briefly, standard curves consisted of ten-fold dilution series of amplified bacterial 16S rRNA genes from reference strains. Samples were amplified with universal primers against total bacteria and specific primers against *Bacteroidaceae*, *Ruminococcaceae*, *Lachnospiraceae*, *Enterobacteriaceae* (Table 1). The abundance of 16S rRNA gene copies was determined from standard curves and specific bacterial groups were expressed as a percentage of total bacteria determined by universal primers. 5 ng of DNA was used per reaction. The same DNA concentration was used for all runs, including universal primer runs which were used to normalize specific bacterial groups against total bacteria, to minimize errors due to any inhibitory substances in the samples. The detection limit was determined with negative controls containing only herring sperm DNA.

**Table 1:** qPCR primers used.

Bacterial family	Primer name	Primer sequence	Reference
All bacteria	UniF	GTGSTGCAYGGYYGTCGTCA	[38]
	UniR	ACGTCRTCCMCNCCTTCCTC	
*Bacteroidaceae*	Bac303F	GAAGGTCCCCCACATTG	
	Bfr-Fmrev	CGCKACTTGGCTGGTTCAG	
*Ruminococcaceae*	Clep866mF	TTAACACAATAAGTWATCCACCTGG	
	Clept1240mR	ACCTTCCTCCGTTTTGTCAAC	
*Lachnospiraceae*	Erec482F	CGGTACCTGACTAAGAAGC	
	Erec870R	AGTTTYATTCTTGCGAACG	
*Enterobacteriaceae*	EnterobactDmod2F	GACCTCGCGAGAGCA	[29]
	Enter1432mod	CCTACTTCTTTTGCAACCCA	

### Bioinformatic and Statistical Analysis

Analysis of sequence data was carried out using the Mothur software package[30]. Initially, the “trim.seqs” function was used to filter reads for quality by truncating them once average quality scores dropped below 35 across a rolling window of 50 bases. In addition all reads with any mismatches to the primer or barcode sequences, plus reads with ambiguous bases (i.e. “N”s) or with homopolymeric stretches of longer than 8 bases were removed. Read length following this step ranged from 336 to 351 bp. Chimeras were then checked for and removed using Perseus software[31], as implemented in Mothur. The sequences were then aligned to the reference SILVA database provided in Mothur, a distance matrix generated, and then clustered into operational taxonomic units (OTUs) at 97% similarity using the average neighbor setting in Mothur. Each OTU was assigned a taxonomic classification at all levels from Phylum to Genus using the reference Ribosomal Database Project database (RDP) provided in Mothur with the *Gemmiger*/*Subdoligranulum* classification error corrected. Jaccard and Yue and Clayton distance matrices were calculated using the vegan package in R[32]. Dendrograms were generated using Ward clustering, and then visualized using the iTOL web package[33].

Comparisons in DNA yield were performed using Mann Whitney U testing. Linear modelling was used to assess the relative contribution of patient, DNA extraction method and extraction site to the measured proportions of different bacterial families. Log-transformed data was used to permit analysis of the fold change. A model was constructed for each bacterial family using the donor source, extraction method and extraction site as covariates. Bacterial families were reported where at least one sample had an abundance of 5% or more. For each family, samples were only included from participants with at least 0.5% abundance for that bacterial family in one or more of their samples. Modelling was also done in a similar manner using individual OTUs. When using linear modelling at the OTU level, Holm's method was used to correct for multiple testing. Correlation between pyrosequencing and qPCR data was done using Pearsons's correlation coefficient. Analysis was performed using R 2.15.2 (R Statistical Foundation, Vienna, Austria).

## Results

**Figure 2:**
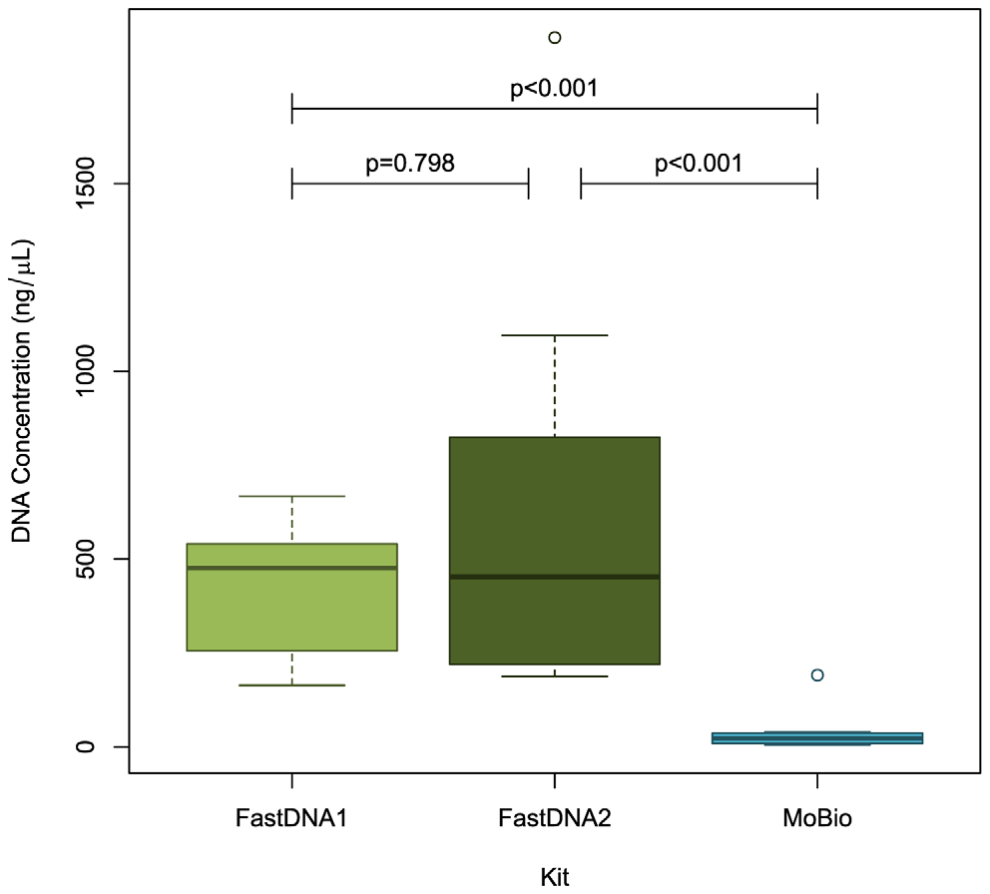
Comparison of DNA yields between extraction methods.

DNA yields were significantly higher with either method of the FastDNA kit than with the MoBio kit, with median DNA concentrations of 476 ng/µL (interquartile range [IQR] 290–519) for FastDNA method 1, 453 ng/µL (IQR 228–689) for FastDNA method 2 and 22 ng/µL (IQR 9-36) for the MoBio method (p<0.001 for both comparisons, Figure 2). There was no significant difference in yield between the two methods of the FastDNA kit (p = 0.798).

Compositional analysis indicated a higher proportion of *Enterobacteriaceae* and *Sutterellaceae*, and lower *Ruminococcaceae*, in the samples from the two IBD patients compared with the two healthy controls, regardless of the extraction method or laboratory. Although this study was clearly not powered to differentiate between IBD cases and controls the higher observed proportions of Proteobacteria in cases, particularly case I2, is consistent with patterns described previously in IBD[6], [7].

**Figure 3:**
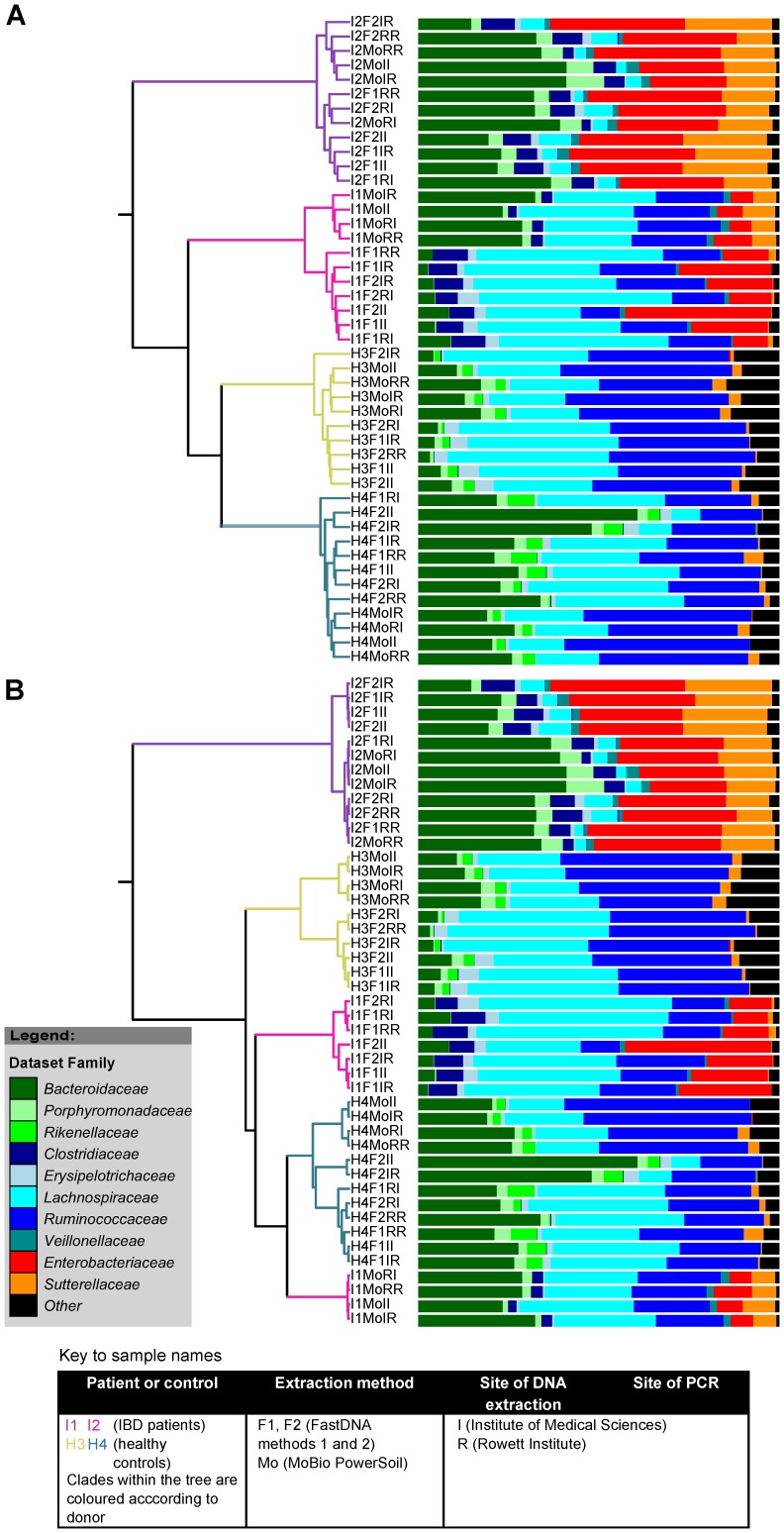
Dendrogram of the representation of bacterial families derived from 16S rRNA gene sequences within each sample clustered by Jaccard (A) and Yue and Clayton (B) distances.

Clustering of the microbiota composition derived from the sequence data for these samples was carried out using both the Jaccard and the Yue and Clayton calculators. The Jaccard calculator is used to describe overlap in community membership between different samples and ignores the proportional abundance of each OTU while, in contrast, the Yue and Clayton calculator takes the proportional abundance of each OTU into account when comparing community similarities. Jaccard-based calculations revealed a clear clustering of samples primarily by subject of origin (Figure 3a). This is as expected given the well-known inter-individual variation in microbiota composition between individuals[34]. Within individuals, however, the MoBio-processed samples tended to cluster together, separately to those processed using the FastDNA kit, indicating that, although there were overall similarities in the range of organisms that were identified using the two DNA extraction kits, there is some bias associated with the use of each kit. More serious repercussions of using different DNA extraction kits were observed when using the Yue and Clayton distance metric, where dominant organisms can have more impact on clustering patterns. The MoBio-processed samples of subject I1 clustered with the samples from subject H4 rather than with the FastDNA-processed samples from patient I1, presumably as a result of elevated *Bacteroides* and lower *Lachnospiraceae* proportional abundances in the MoBio extractions compared to the FastDNA extractions (Figure 3b). This demonstrates that biases introduced by DNA extraction methodology can over-ride the real underlying patterns of community structure driven by inter-individual variation.

Linear modelling of the family level data for the top nine families represented in the pyrosequencing data is shown in Table 2 (range of abundances in table S2). Significant differences were identified between the FastDNA and MoBio kits, with relatively higher *Bacteroidaceae*, *Ruminococcaceae* and *Porphyromonadaceae*, and lower *Enterobacteriaceae, Lachnospiraceae*, *Clostridiaceae* and *Erysipelotrichaceae* following extraction with the MoBio kit. There was a significant difference identified between the two methods of the FastDNA kit in just one family, the *Rikenellaceae*. The extraction site made a significant difference only for *Sutterellaceae*, with the observed differences being driven by an increase in one OTU in samples from patients H4 and I1 when they had been extracted at RINH (Using FastDNA methods in patient H4, relative abundance 0.24% (95% confidence interval 0.11-0.38%) at IMS and 2.64% (0.85-4.44%) at RINH.) The site at which the amplification PCR was performed made no significant difference for any of the bacterial families analyzed, and was therefore excluded from the models.

**Table 2:** Linear modelling of family-level pyrosequencing data.

Bacterial Family	Kit				Extraction Site		
	FastDNA 2 fold change	p	MoBio fold change	P	RINH fold change	p	Patients included
*Lachnospiraceae*	0.96 (0.74–1.25)	0.775	0.63 (0.49–0.81)	0.001	1.17 (0.95–1.44)	0.160	H3,H4,I1,I2
*Bacteroidaceae*	1.13 (0.79–1.63)	0.501	2.13 (1.49–3.05)	<0.001	1.09 (0.81–1.46)	0.561	H3,H4,I1,I2
*Ruminococcaceae*	0.94 (0.79–1.13)	0.524	1.32 (1.11–1.58)	0.005	0.95 (0.82–1.10)	0.516	H3,H4,I1
*Enterobacteriaceae*	1.08 (0.74–1.57)	0.695	0.61 (0.43–0.88)	0.016	0.85 (0.63–1.15)	0.311	I1,I2
*Sutterellaceae*	0.77 (0.18–3.37)	0.735	1.11 (0.26–4.69)	0.892	3.84 (1.18–12.46)	0.031	H3,H4,I1,I2
*Clostridiaceae*	1.00 (0.77–1.30)	0.976	0.46 (0.36–0.59)	<0.001	0.88 (0.71–1.08)	0.243	I1,I2
*Porphyromonadaceae*	1.46 (0.41–5.19)	0.560	4.03 (1.16–14.01)	0.035	0.70 (0.26–1.94)	0.502	H3,H4,I1,I2
*Erysipelotrichaceae*	1.21 (0.81–1.81)	0.361	0.32 (0.21–0.47)	<0.001	0.88 (0.64–1.22)	0.445	H3,H4,I1,I2
*Rikenellaceae*	0.35 (0.16–0.76)	0.016	0.72 (0.33–1.56)	0.418	0.65 (0.35–1.19)	0.181	H3,H4

RINH: Rowett Institute of Nutrition and Health.

Participants were excluded if all data points for that bacterial family were < 0.5%. Reference sample was from participant H3 using FastDNA method 1 and extracted at the Institute of Medical Sciences. Differences are shown as fold change with 95% confidence intervals.

At the OTU level, 18 OTUs were significantly different between the MoBio kit and the FastDNA kit after correction for multiple testing (Table 3). Of these, 10 were from the *Lachnospiraceae* family and 8 of these 10 were relatively under-represented in the MoBio processed samples, in some cases with a complete absence of the OTU in the MoBio samples.

**Table 3:** Multiple linear modelling after correction for multiple testing shows OTUs with significantly different relative abundance after extraction with the MoBio kit.

Genus	Family	Order	Class	Phylum	Fold change	p	Corrected p	Patients included
***Eggerthella***	*Coriobacteriaceae*	Coriobacteriales	Actinobacteria	Actinobacteria	0.00 (0.00–0.00)	5.09×10^−9^	5.55×10^−7^	I1
***Blautia***	*Lachnospiraceae*	Clostridiales	Clostridia	Firmicutes	0.00 (0.00–0.01)	1.70×10^−7^	1.83×10^−5^	H3,H4,I1
***Blautia***	*Lachnospiraceae*	Clostridiales	Clostridia	Firmicutes	0.01 (0.00–0.04)	2.01×10^−7^	2.15×10^−5^	H3,H4,I1
***Bacteroides***	*Bacteroidaceae*	Bacteroidales	Bacteroidia	Bacteroidetes	2.60 (1.92–3.52)	3.26×10^−7^	3.46×10^−5^	H3,H4,I1,I2
***Clostridium sensu stricto***	*Clostridiaceae*	Clostridiales	Clostridia	Firmicutes	0.36 (0.30–0.43)	3.91×10^−6^	0.0004	I1
***Blautia***	*Lachnospiraceae*	Clostridiales	Clostridia	Firmicutes	0.01 (0.00–0.05)	1.97×10^−5^	0.0020	H3,H4,I1
**unclassified**	*Ruminococcaceae*	Clostridiales	Clostridia	Firmicutes	0.17 (0.12–0.24)	3.13×10^−5^	0.0032	H3
**unclassified**	*Lachnospiraceae*	Clostridiales	Clostridia	Firmicutes	0.00 (0.00–0.01)	3.49×10^−5^	0.0036	H4,I1
***Anaerostipes***	*Lachnospiraceae*	Clostridiales	Clostridia	Firmicutes	0.10 (0.04–0.23)	5.23×10^−5^	0.0053	H3,H4
***Escherichia Shigella***	*Enterobacteriaceae*	Enterobacteriales	Gamma-proteobacteria	Proteobacteria	0.41 (0.28–0.59)	1.36×10^−4^	0.0136	I1,I2
**unclassified**	*Lachnospiraceae*	Clostridiales	Clostridia	Firmicutes	5.05 (2.55–9.97)	2.24×10^−4^	0.0222	H3,I1
***Ruminococcus***	*Ruminococcaceae*	Clostridiales	Clostridia	Firmicutes	0.48 (0.34–0.68)	0.0002	0.0232	H3,H4,I1
**unclassified**	*Lachnospiraceae*	Clostridiales	Clostridia	Firmicutes	2.50 (1.92–3.26)	0.0003	0.0250	H3
**unclassified**	*Ruminococcaceae*	Clostridiales	Clostridia	Firmicutes	3.19 (2.26–4.50)	0.0003	0.0298	H3
***Bacteroides***	Bacteroidaceae	Bacteroidales	Bacteroidia	Bacteroidetes	2.03 (1.42–2.89)	0.0004	0.0342	H3,H4,I1,I2
***Dorea***	Lachnospiraceae	Clostridiales	Clostridia	Firmicutes	0.00 (0.00–0.06)	0.0004	0.0343	H3,H4,I1
**unclassified**	Lachnospiraceae	Clostridiales	Clostridia	Firmicutes	0.00 (0.00–0.00)	0.0004	0.0358	I1
***Dorea***	Lachnospiraceae	Clostridiales	Clostridia	Firmicutes	0.06 (0.01–0.25)	0.0004	0.0381	H3,H4,I1,I2

Samples were excluded if all data points for that bacterial family were <0.5%. Reference sample was from patient H3 using either FastDNA method and extracted at the Institute of Medical Sciences. Differences are shown as fold change with 95% confidence intervals.

**Figure 4:**
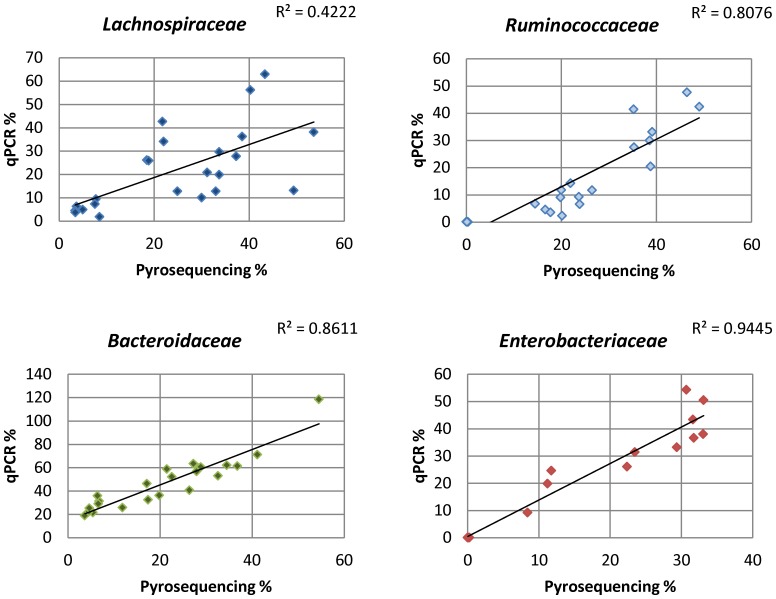
Correlation between pyrosequencing and qPCR data.

Correlation between pyrosequencing and qPCR data was generally good (Figure 4), with R^2^ values of 0.81, 0.86 and 0.94 for *Ruminococcaceae*, *Bacteroidaceae* and *Enterobacteriaceae* respectively. However, the correlation was less good for the *Lachnospiracaeae*, with an R^2^ value of 0.42. Linear modelling revealed similar differences to that seen in the pyrosequencing data, although the differences related to extraction method were only significant for *Ruminococcaceae* and *Enterobacteriaceae* (Tables 4, S3).

**Table 4:** Linear modelling of qPCR data.

Bacterial Family	Kit				Extraction Site		
	FastDNA 2 fold change	p	MoBio fold change	P	RINH fold change	p	Patients included
*Lachnospiraceae*	0.75 (0.48–1.17)	0.209	0.74 (0.48–1.16)	0.199	1.35 (0.94–1.94)	0.107	H3,H4,I1,I2
*Bacteroidaceae*	1.19 (0.94–1.51)	0.147	1.25 (0.99–1.57)	0.066	0.98 (0.81–1.18)	0.822	H3,H4,I1,I2
*Ruminococcaceae*	0.69 (0.47–1.01)	0.070	2.32 (1.58–3.39)	<0.001	1.26 (0.92–1.71)	0.157	H3,H4,I1
*Enterobacteriaceae*	1.28 (0.97–1.69)	0.102	0.65 (0.48–0.87)	0.011	0.91 (0.72–1.15)	0.436	I1,I2

RINH: Rowett Institute of Nutrition and Health.

Participants were excluded if all data points for that bacterial family were <0.5%. Reference sample was from participant H3 using FastDNA method 1 and extracted at the Institute of Medical Sciences. Differences are shown as fold change with 95% confidence intervals.

## Discussion

With the recognition that cultured bacteria cover only a small proportion of gut microbial diversity[35], a number of molecular techniques have been developed to describe and quantify the gut microbiota, from qualitative gel-based methods to full metagenomic sequencing[5], [36]. Almost all of these techniques require extraction of DNA from fecal or mucosal samples as a first step, and differences at this point will influence downstream results. The importance of this will be amplified if, for example, cases and controls are processed in a different manner.

This study highlights important differences in the performance of two commercially available kits for DNA extraction from fecal samples. Significantly lower DNA yields were seen with the MoBio kit than the FastDNA kit. This is consistent with results published previously[19]. More importantly, there were significant differences in the relative abundance of bacteria measured at both the family and OTU level. There is no gold standard to which these data can be compared, and so it is impossible to say which technique yields results closer to the true profile of the samples. However, the lower yield of the MoBio kit, and reduced proportional abundance of the *Lachnospiraceae* family of Firmicutes, suggests that this kit may not be stringent enough for optimal lysing of some Gram-positive organisms. Regardless, these differences are such that it is important to stipulate that all samples in a particular experiment should be extracted using the same technique. This is of particular importance with multicenter studies. Moreover, it should prompt researchers to exercise caution when comparing datasets from different studies. Indeed, if DNA has been extracted using different kits then studies should not be considered cross-comparable. Of note, a recent meta-analysis found that samples from studies of fecal microbiota within Western populations clustered by study, suggesting that systematic bias was introduced by factors such as DNA extraction technique[37].

The importance of the observed differences will depend on the analysis techniques used. However, whenever a relative quantification technique is used, the results for even a single organism will be influenced by the effects of the extraction technique on the total number of bacteria isolated relative to that specific species. The methods for both kits used here involved physical disruption by bead-beating. Methods that rely on enzymatic treatment without physical disruption have been shown previously to give biased recovery, with reduced recovery of Gram-positive organisms and artificially elevated levels of Gram negatives, presumably because these are more easily lysed[18], [22].

A smaller effect was observed of the extraction site on relative abundance, with only *Sutterellaceae* reaching statistical significance. This may reflect a difference in operator, equipment or laboratory environment. To minimize the influence of differences between laboratories, centralization of DNA extraction for an experiment would be preferred. The technique described here includes only minimal processing after sample collection prior to interim storage at −80°C. This allows for collection sites to collate a number of samples in −80°C storage prior to shipment to a central facility for DNA extraction and downstream analysis.

The qPCR data in general correlated well with that from pyrosequencing with the exception of *Lachnospiracaeae*. This can be partially explained by differences between the range of organisms that were targeted by the qPCR primers and those that were classified as *Lachnospiracaeae* in the pyrosequencing data, although 78% of OTUs and 89% of sequences labelled as *Lachnospiracaeae* were estimated *in silico* to be targeted by the qPCR primer set used.

Ariefdjohan et al. previously assessed the effect of DNA extraction method on the measured bacterial composition of stool using denaturing gradient gel electrophoresis (DGGE)[19]. This study demonstrated variability in bacterial community between fecal samples extracted with QIAamp DNA, MoBio Ultra Clean Fecal DNA and FastDNA SPIN kits, and noted that both the MoBio and Qiagen kits were not able to extract DNA from all the bacteria in the specimen. More recently, Claassen et al. used DGGE, terminal restriction fragment length polymorphism (T-RFLP) and qPCR to compare fecal samples extracted using kits from Qiagen, ZymoResearch and MoBio and found few significant differences[20]. In contrast, the previous study by Wu *et al.* which assessed the effect of extraction methods on 16S rRNA pyrosequencing demonstrated increased yield of Firmicutes when a hot phenol bead-beating method or the PSP kit were used. The present study helps bring further clarity to this important issue with next generation sequencing permitting a more detailed exploration of the differences between samples extracted with different methods.

This study is somewhat limited by its relatively small sample size, with fecal samples obtained from only four individuals. There were a small number of outliers; samples H4F2AA and H4F2AR had much higher relative abundance of *Bacteroidaceae*. In addition, the data obtained here from both pyrosequencing and qPCR estimate relative abundance rather than absolute numbers and focus on the dominant groups within the microbiota.

## Conclusions

This study demonstrates important differences in the yield and relative abundance of key bacterial families for kits used to isolate bacterial DNA from stool. This highlights the importance of ensuring that all samples to be analyzed together are prepared with the same DNA extraction method, and the need for caution when comparing studies that have used different methods.

## Supporting Information

Table S1 Barcodes used for each sample included in the study, and the respective ENA-deposited dataset that they can be recovered from.
(XLSX)

Table S2 Relative abundances of the top nine bacterial families measured for each individual.
(DOCX)

Table S3 Relative abundances of the bacterial families measured by quantitative PCR for each individual.
(DOCX)
