# Supplementary material for: The Impact of Different DNA Extraction Kits and Laboratories upon the Assessment of Human Gut Microbiota Composition by 16S rRNA Gene Sequencing
Source: PLoS One. 2014 Feb 24;9(2):e88982. doi: 10.1371/journal.pone.0088982 (PMC3933346; doi:10.1371/journal.pone.0088982)
Supplement: Table S2 — Relative abundances of the top nine bacterial families measured for each individual. (DOCX) [file pone.0088982.s002.docx]

Table S2: Relative abundances of the top nine bacterial families measured for each individual

| Bacterial Family | **Relative abundance median (range [%])** | | | |
| --- | --- | --- | --- | --- |
|  | **H3** | **H4** | **I1** | **I2** |
| *Lachnospiraceae* | 33.00 (18.93-44.72) | 24.41 (7.99-38.91) | 37.53 (24.10-53.59) | 4.49 (2.50-8.95) |
| *Bacteroidaceae* | 7.82 (3.36-17.46) | 26.37 (19.18-60.88) | 8.54 (2.69-32.45) | 32.61 (14.79-41.21) |
| *Ruminococcaceae* | 38.75 (31.33-47.65) | 25.21 (17.06-51.47) | 18.77 (10.86-24.51) | 0.11 (0.00-0.46) |
| *Enterobacteriaceae* | 0.05 (0.00-0.33) | 0.00 (0.00-0.18) | 11.73 (6.12-40.43) | 29.34 (21.23-37.40) |
| *Sutterellaceae* | 1.58 (0.30-3.86) | 1.05 (0.00-5.50) | 1.40 (0.07-9.06) | 14.83 (9.90-24.16) |
| *Clostridiaceae* | 0.08 (0.03-0.33) | 0.09 (0.00-0.22) | 7.04 (2.36-9.66) | 6.24 (2.57-9.33) |
| *Porphyromonadaceae* | 2.05 (0.33-4.15) | 2.86 (1.17-4.58) | 0.16 (0.00-2.76) | 4.52 (2.71-10.50) |
| *Erysipelotrichaceae* | 2.68 (0.65-5.67) | 1.18 (0.25-4.31) | 2.41 (0.48-5.87) | 1.31 (0.13-2.66) |
| *Rikenellaceae* | 2.54 (0.18-3.00) | 3.49 (0.37-7.56) | 0.00 (0.00-0.09) | 0.00 (0.00-0.34) |
