# Supplementary material for: The Impact of Different DNA Extraction Kits and Laboratories upon the Assessment of Human Gut Microbiota Composition by 16S rRNA Gene Sequencing
Source: PLoS One. 2014 Feb 24;9(2):e88982. doi: 10.1371/journal.pone.0088982 (PMC3933346; doi:10.1371/journal.pone.0088982)
Supplement: Table S3 — Relative abundances of the bacterial families measured by quantitative PCR for each individual. (DOCX) [file pone.0088982.s003.docx]

Table S3: Relative abundances of the bacterial families measured by quantitative PCR for each individual

| Bacterial Family | **Relative abundance median (range [%])** | | | |
| --- | --- | --- | --- | --- |
|  | **H3** | **H4** | **I1** | **I2** |
| *Lachnospiraceae* | 42.69 (29.64-63.00) | 23.33 (1.83-27.76) | 12.78 (10.06-38.12) | 5.59 (3.75-9.43) |
| *Bacteroidaceae* | 25.74 (21.45-32.36) | 59.21 (36.26-118.38) | 35.85 (18.90-60.49) | 57.19 (46.37-71.11) |
| *Ruminococcaceae* | 33.11 (20.44-47.67) | 10.49 (2.29-42.40) | 9.06 (3.55-14.27) | 0.05 (0.05-0.07) |
| *Enterobacteriaceae* | 0.02 (0.01-0.04) | 0.01 (0.00-0.01) | 24.58 (9.19-33.23) | 40.73 (26.08-54.22) |
